# Reduced serum levels of anti-Mullerian hormone is a putative biomarker of early knee osteoarthritis in middle-aged females at menopausal transition

**DOI:** 10.1038/s41598-021-84584-0

**Published:** 2021-03-02

**Authors:** Eiji Sasaki, Daisuke Chiba, Seiya Ota, Yuka Kimura, Shizuka Sasaki, Yuji Yamamoto, Maika Oishi, Kaori Iino, Masataka Ando, Eiichi Tsuda, Yasuyuki Ishibashi

**Affiliations:** 1 Department of Orthopedic Surgery, Hirosaki University Graduate School of Medicine, 5 Zaifu-cho, Hirosaki, Aomori 036-8562 Japan; 2 Department of Obstetrics and Gynecology, Hirosaki University Graduate School of Medicine, Hirosaki, Japan; 3 Department of Social Medicine, Hirosaki University Graduate School of Medicine, Hirosaki, Japan; 4 Department of Rehabilitation Medicine, Hirosaki University Graduate School of Medicine, Hirosaki, Japan

**Keywords:** Predictive markers, Ageing, Bone

## Abstract

A recent epidemiological study revealed that the highest prevalence of early knee osteoarthritis (OA) was observed in females aged ≥ 50 years. The major causal factor of early knee OA was sex. Despite the relevance of estrogen in evaluating chondral and bone metabolism in OA, it is not easily clinically monitored because irregular menstrual cycles induce unstable female hormone patterns during menopausal transitions. Anti-Mullerian hormone (AMH) has been found to be a new stable biomarker to predict menopause. This study aimed to investigate the association between menopausal transition and early knee OA by using serum biomarkers, with special focus on AMH. A total of 518 female volunteers who participated in the Iwaki cohort study were enrolled and divided into pre-menopause and post-menopause groups. Weight-bearing anterior–posterior knee radiographs were classified by Kellgren–Lawrence (KL) grade, and grade ≥ 2 was defined as radiographic knee OA. In participants with KL grades 0 and 1, early knee OA was defined by Luyten’s criteria. AMH, luteinizing hormone, follicle-stimulating hormone, estradiol (pg/ml), prolactin, and testosterone were measured on the female hormones. Bone mineral density at a distal radius was measured. The predictive power of female hormones for early knee OA was estimated by ROC analysis (comparison of area under curve, AUC) and regression analysis. Fifty-two participants (10.0%) were diagnosed with early knee OA and 204 (39.4%) with radiographic knee OA. In 393 (75.9%) females, menopause began. From the ROC analysis in pre-menopausal females, cutoff value of AMH for detecting early knee OA was 0.08 ng/ml (area under curve (AUC), 0.712; 95% CI, 0.527–0.897; *p* value, 0.025; odds ratio, 8.28). AUCs of other female hormones did not reach the level of AMH (range, 0.513 of prolactine to 0.636 of estradiol). Logistic regression analysis focusing on AMH reduction at menopausal transition showed that the related AMH below 0.08 ng/ml was significantly related to the presence of early knee OA (*p* = 0.035; odds ratio, 5.55). Reduced serum levels of AMH in middle-aged females were correlated with the presence of early knee OA, which might be a useful serum biomarker.

## Introduction

Knee osteoarthritis (OA) is a common cause of chronic pain and disability in elderly people^1^, and its radiographic prevalence among adults aged > 40 years in Japan was 42% in males and 62.4% in females^2^. Progressed knee OA causes high treatment costs, decreased productivity, and absence from work^3^; therefore, early diagnosis is crucial for early treatment and preventative interventions^4^. Recently, the concept and diagnostic criteria for early knee OA was established^4^, and a current epidemiological study revealed that the highest prevalence of early knee OA was observed in females aged 50 years old^5^.

In these population, female sex is a major causal factor of knee OA^6,7^. Its prevalence in females were 1.2–2.8 times higher than that in males^5,8–12^, and cartilage loss progressed more rapidly in females^13^. Estrogen deficiency after menopause is known to cause a reduction in bone mineral density (BMD)^14^ and suppress cartilage and subchondral bone remodeling^15,16^. However, despite the relevance of estrogen in evaluating chondral and bone metabolism, few reports exist to investigate the relationship between decreasing female hormones and early knee OA, whereas both conditions are observed among females ≥ 50 years old. One of the reasons is that the female hormones are not easily clinically monitored because they are in menopausal transitions. Menopausal transition is pre-menopausal condition and encompasses a period of dynamic changes in reproductive and nonreproductive tissues, which causes irregular menstrual cycles in middle-aged females^17^.

Thus, it is difficult to fully understand the relationship between dysfunction of female hormones and early knee OA. In contrast, recently, anti-Mullerian hormone (AMH) has been found to be a new stable biomarker to predict menopause. AMH is a glycoprotein dimer, only expressed in growing follicles, and has been identified as a marker of ovarian aging^18,19^. Moreover, serum levels of AHM decreases during menopausal transition, as previous reports found that the serum levels of AMH reduces 3 years before menopause^20^. Another advantage of AMH is that it is not affected by menstrual cycles like estradiol, luteinizing hormone (LH), and follicle-stimulating hormone (FSH). Hence, the serum levels of AMH are beneficial in understanding the association of follicle activity during the early phase of knee OA.

This epidemiological study aimed to investigate the association between menopausal transition and early knee OA by using several serum biomarkers, with special focus on AMH. Furthermore, the influence of bone fragility related to menopause on knee OA was investigated. We hypothesized that reduced serum levels of AMH could sensitively reflect the presence of early knee OA. Furthermore, progressed stage of knee OA would be associated with menopause and bone fragility regardless of serum levels of AMH.

## Methods

The participants were volunteers for the Iwaki Health Promotion Project, which is a community-based preventive medicine program that aims at improving average life expectancy by performing general health check-ups^5,21,22^. The participants were recruited using mass media advertisements and with the help of public health nurses. All participants provided written informed consent, and the study was done in agreement with the 1964 Helsinki Declaration and its later amendments or comparable ethical standards and conducted with the approval of the ethics committee of Hirosaki University Graduate School of Medicine (2016-028).

### Participants

**Figure 1:**
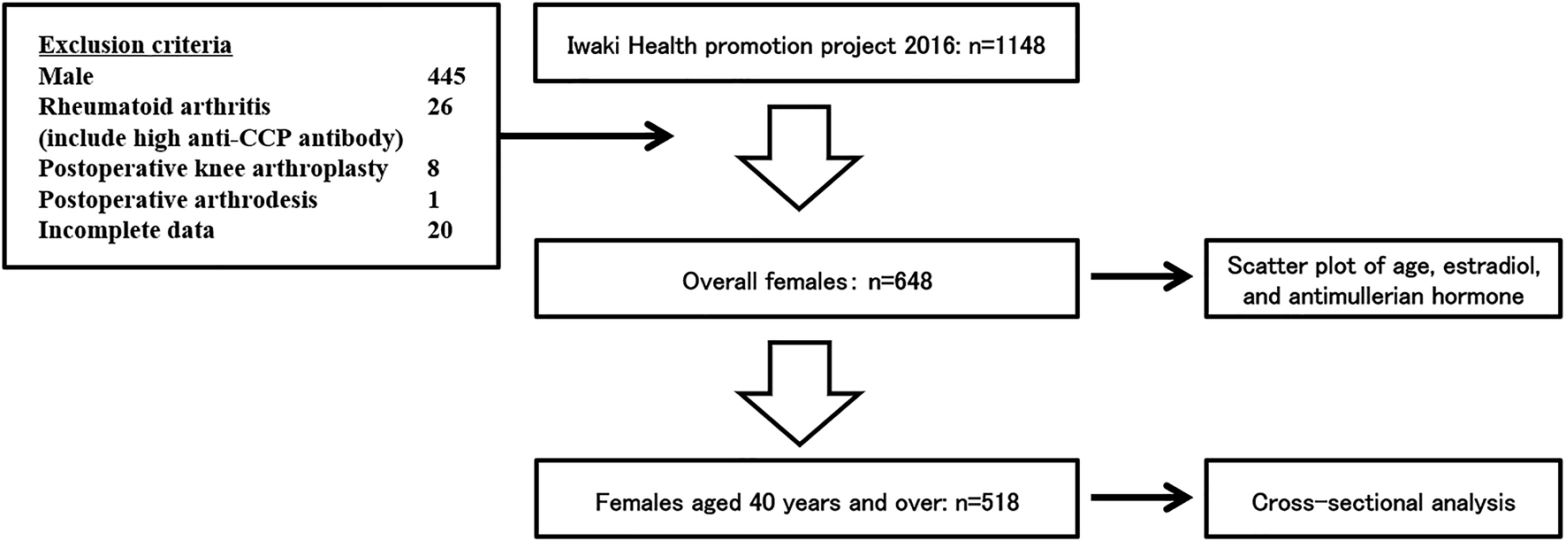
Flow of participants enrollment in Iwaki Health promotion project. Finally, 518 females were enrolled for the statistical analysis.

**Figure 2:**
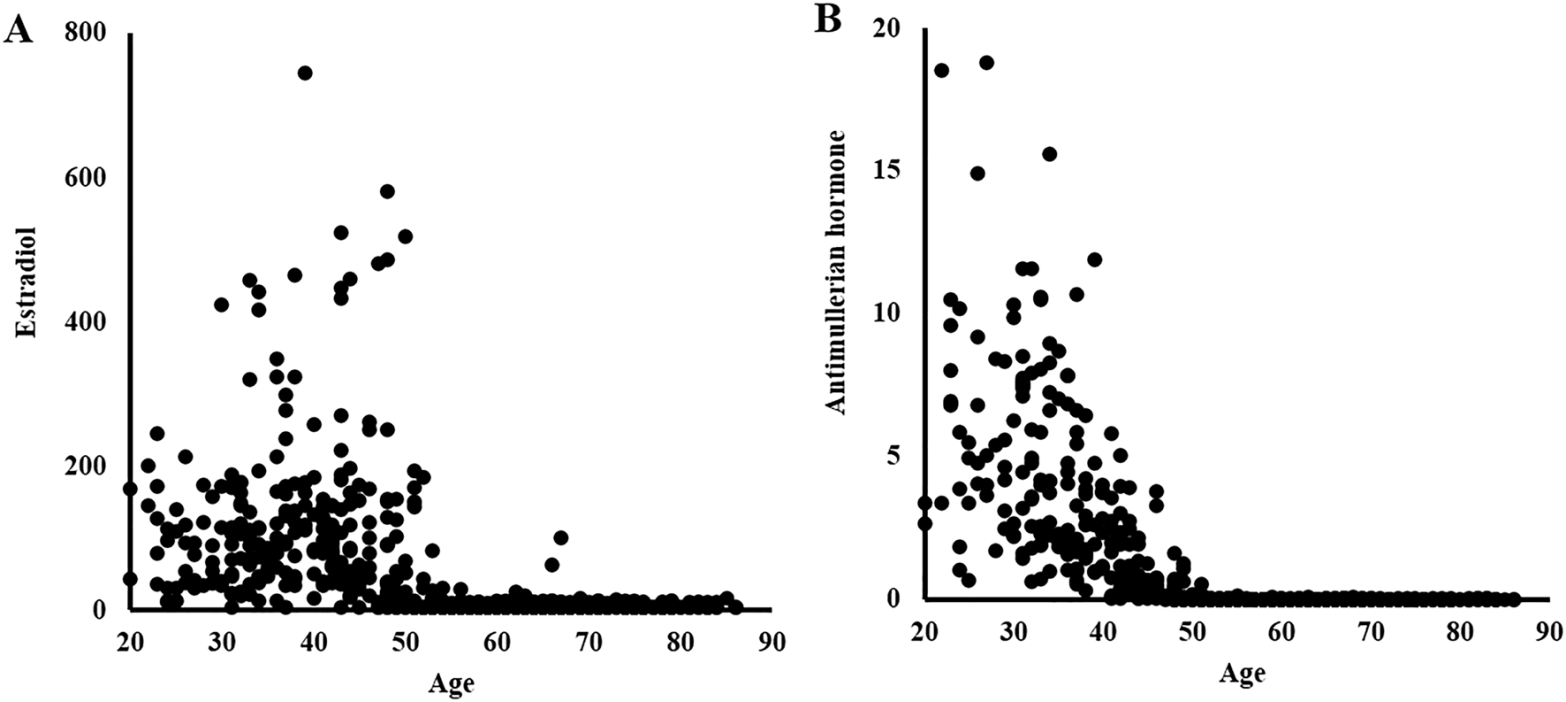
Scatter plot between age and female hormone levels. The distribution of serum estradiol (**A**) and AMH (**B**) per their ages were shown in a scatter plot.

Out of the 12,000 eligible residents, 1148 volunteers were enrolled in this study (Fig. 1). The exclusion criteria of this study were as follows: male sex (n = 455), patients under treatment of rheumatoid arthritis or with anti-cyclic citrullinated peptide antibody of more than 4.5 U/ml (n = 26), postoperative patients with total knee arthroplasty and arthrodesis (n = 9), and incomplete data (n = 20). After the first exclusion, a total of 648 female participants were included, and their distribution of estradiol (Fig. 2A) and AMH (Fig. 2B) per their ages were shown in a scatter plot. Data of females aged 40 years and above were considered for further statistical analysis. All the participants answered self-reported questionnaires about menstrual condition, and the menopause was defined as the time when there has been no menstrual periods for 12 consecutive months. Additional questionnaires concerning lifestyle habits, including routine alcohol consumption, cigarette smoking, and fitness habits, were administered. The presence of these habits was defined as routine drinking 1 or more days a week regardless of the amount of alcohol, smoking more than a cigarette every day, or performing exercise ≥ 2 days per week regardless of fitness type and length. Former habits and chance drinking were not included for lifestyle habits. These habits were included as categorical variables (yes/no) in the statistical analysis. For the anthropometric evaluation, height and weight were measured and recorded and body mass index (BMI) was calculated.

### Blood examination

Blood samples were taken from all participants before breakfast after a period of 10 or more hours without food. Blood analysis were performed by the LSI Medience Corporation (LSI Medience Corp., Tokyo, Japan). This company has an ISO-15189-accredited laboratory, and serum assays were performed under strict conditions. AMH (ng/ml, CLEIA), LH (mIU/ml, CLIA), FSH (mIU/ml, CLIA), estradiol (pg/ml, CLIA), prolactin (ng/ml, CLIA), and testosterone (ng/ml, CLIA) were measured on the female hormones. Further, bone metabolism was evaluated by the following markers: type I procollagen N-terminal propeptide total (total PINP, μg/l, ECLIA), bone alkaline phosphatase (BAP, μg/l, CLEIA), N-terminal telopeptide (NTx, nMBCE/l, EIA), and tartrate-resistant acid phosphatase 5b (TRACP-5b, mU/dl, EIA). Furthermore, pentosidine (pmol/ml, HPLC) and homocysteine (nmol/ml, LC–MS/MS) were measured for bone quality. The inflammation markers hyaluronan (ng/ml, LA), matrix metalloproteinase-3 (MMP-3, ng/ml, LA), high sensitivity C-reactive protein (hr-CRP, mg/dl, Nephelometry), adiponectin (μg/ml, LA), leptin (ng/ml, RIA), and interleukin-6 (IL-6, pg/ml, CLEIA) were examined.

### Radiographic evaluation

A radiographic examination of the knee was performed using a digital radiographic device (CXDI-40EG, Canon Inc. Tokyo, Japan). Experienced radiologists and orthopedic surgeons obtained the weight-bearing, full extension, and anterior–posterior radiographs of both knees with foot map positioning on the day of the check-up. The beam was positioned parallel to the floor and aimed at the joint space, and the sequencing was set at 60 kV, 50 mAs, and 80 ms for all participants. The images were converted into JPEG format files. OA severity in each knee was classified as KL grade 0 to 4 using the KL radiographic atlas by two trained orthopedic surgeons (DC and SO)^23^. The interclass correlation coefficient from the two surgeons was 0.815. These surgeons were blinded to the sequence in which the radiographs were acquired and the clinical status of the participants. In this study, KL grades 0 and 1 were classified into pre-radiographic OA stage, non-OA, and early knee OA. Participants with KL grades 2, 3, or 4 in the most affected knees were diagnosed with radiographic knee OA.

### Classification criteria for early OA of the knee

Early knee OA was defined based on the classification criteria proposed by Luyten^4^. The classification criteria for early knee OA were as follows: A) patient-based questionnaires, the knee injury and osteoarthritis outcome scores (KOOS)^24^ (two of the following needed to score “positive” (i.e., ≤ 85%): pain (9 items), symptoms (7 items), activities of daily living (ADL) (short version, 7 items), and knee-related quality of life (QOL) (4 items)); B) clinical examination (at least one of the following needed to present: joint line tenderness or crepitus of the knee); C) radiographs, KL grade zero and one at the standing, extension, and weight-bearing positions. Based on the above criteria, subjects with KL grade 0/1 were classified into non-OA and early knee OA.

### Bone mineral density measurements

The BMD of the forearm was determined by dual-energy X-ray absorptiometry using DCS-600EXV (Hitachi Aloka Medical, Tokyo, Japan) based on a previous report^25^. Briefly, the region of interest of the BMD was measured on the non-dominant side, one third of the distal radius, unless there was a history of previous fracture, where the dominant side was measured.

### Statistical analysis

Demographic data among non-OA, early knee OA, and radiographic knee OA groups were shown as mean ± standard deviations. Chi-square test for categorical variables and analysis of variance (ANOVA) and Tukey test for continuous variables were performed to compare the three groups’ demographic data. To estimate the odds ratio of radiographic knee OA by menopause, crude logistic regression analysis was performed, with radiographic knee OA as a dependent variable and menopause as an independent variable. To estimate cutoff values of female hormones for early knee OA among pre-menopausal females, receiver operating characteristic (ROC) analysis was performed. The plot of false-positive fraction and true-positive fraction build curve and area under the curve (AUC) was calculated. The cutoff point was defined from the nearest point to the true positive. Based on the cutoff value of AMH and menopausal condition, the prevalence of early knee OA and radiographic knee OA were estimated. Furthermore, the Spearman’s correlation coefficients were calculated using female hormones, bone metabolic markers, and inflammation markers. Finally, to investigate the correlation among menopause, AMH, early knee OA, and radiographic knee OA, logistic regression analyses using a backward stepwise selection method were performed, with the early knee OA or radiographic knee OA as a dependent variable and BMI, BMD, bone metabolic markers, and inflammation markers as basal independent variables. In addition to basal independent variables, regression model 1 was set to investigate the risk of lower AMH on early knee OA in pre-menopausal females with KL grade 0 and 1. In this analysis, categorized AMH (pre-menopause with AMH ≥ 0.08, pre-menopause with AMH < 0.08, or post-menopause) was used as an independent variable. Subsequently, the same independent variables were used in regression model 2 to investigate the risk of radiographic knee OA. Regression model 3 included a categorized post-menopausal period of 5 years. Data input and analysis were performed using SPSS version 25.0 J (SPSS Inc., Chicago, IL, USA). A *p* value < 0.05 was considered statistically significant.

### Ethics approval and consent to participate

All participants provided written informed consent, and the study was done in agreement with the 1964 Helsinki Declaration and its later amendments or comparable ethical standards and conducted with the approval of the ethics committee of Hirosaki University Graduate School of Medicine (2016-028).

### Consent for publication

Consent for publication were obtained from all of participants with consent to participate.

## Results

### Demographics of participants

Out of the 518 participants, 262 (50.6%) were classified as non-OA, 52 (10.0%) as early knee OA, and 204 (39.4%) as radiographic knee OA. There were no significant differences of age, BMI, and BMD between non-OA and early knee OA groups (Table 1). A total of 393 (75.9%) females were 49.4 ± 4.7 years old. The prevalence of OA in post-menopausal women was up to 48.1% and 12.0% in the pre-menopausal participants (*p* < 0.001). Regression analysis revealed that the odds ratio of radiographic knee OA by menopause was 6.79 (*p* < 0.001; 95% CI, 3.82–12.07).

**Table 1:** Demographic data of non-KOA, early knee OA, and radiographic knee OA groups.

	Non-OA	Early knee OA	Radiographic knee OA
Sample number	262	52	204
Age (y.o)	55.8 ± 10.5	58.8 ± 7.8	67.2 ± 9.8*†
Post-menopause (%)	161 (61.5%)	43 (82.7%)	189 (92.6%) *
Body mass index (kg/m^2^)	22.0 ± 2.9	22.8 ± 3.5	23.8 ± 3.5*
Bone mineral density (g/cm^2^)	0.59 ± 0.10	0.58 ± 0.09	0.52 ± 0.09*†
KOOS pain	97.3 ± 6.0	75.4 ± 13.8*	79.5 ± 20.0*
KOOS symptom	95.6 ± 5.6	78.7 ± 12.8*	80.2 ± 19.7*
KOOD ADL short	97.5 ± 7.0	79.2 ± 16.1*	80.2 ± 21.6*
KOOS QOL	89.9 ± 14.3	58.2 ± 16.7*	63.2 ± 27.2*
Anti-Mullerian hormone (ng/ml)	0.39 ± 0.88	0.08 ± 0.31*	0.08 ± 0.46*
Estradiol (pg/ml)	50.9 ± 95.7	19.6 ± 50.8*	11.0 ± 21.0*
Luteinizing hormone (mIU/ml)	16.9 ± 11.2	19.6 ± 7.2	18.5 ± 9.2
Follicle-stimulating hormone (mIU/ml)	42.5 ± 29.8	53.9 ± 23.7*	50.9 ± 20.6*
Prolactin (ng/ml)	10.0 ± 9.2	10.6 ± 13.1	10.5 ± 16.2
Testosterone (ng/ml)	0.24 ± 0.09	0.24 ± 0.15	0.24 ± 0.09
Type I procollagen N-terminal propeptide total (μg/l)	49.2 ± 24.7	54.9 ± 20.6	52.6 ± 22.5
Bone alkaline phosphatase (μg/l)	13.4 ± 6.2	14.8 ± 4.6	15.6 ± 8.3*
N-terminal telopeptide (nMBCE/l)	15.0 ± 4.6	16.9 ± 4.7	16.7 ± 6.5*
Tartrate-Resistant Acid Phosphatase 5b (mU/dl)	424.7 ± 194.6	508.8 ± 200.9*	509.7 ± 193.9*
Pentosidine (pmol/ml)	27.7 ± 11.9	30.4 ± 11.2	33.4 ± 16.3*
Homocysteine (nmol/ml)	8.2 ± 2.7	8.3 ± 1.9	9.0 ± 3.3*
Hyaluronic acid (ng/ml)	26.3 ± 29.7	30.7 ± 24.5	90.7 ± 262.6*
Matrix metalloproteinase-3 (ng/ml)	35.3 ± 14.3	39.7 ± 29.7	44.7 ± 34.5*
High sensitivity C-reactive protein (mg/dl)	0.06 ± 0.14	0.05 ± 0.14	0.09 ± 0.18
Adiponectin (μg/ml)	12.5 ± 6.0	12.1 ± 5.0	13.2 ± 6.1
Leptin (ng/ml)	10.0 ± 5.7	10.3 ± 5.8	12.2 ± 8.5*
Interleukin-6 (pg/ml)	1.3 ± 1.6	2.2 ± 6.1	1.9 ± 2.2*
Fitness habit (%)	58 (22.1%)	13 (25.0%)	48 (23.5%)
Drinking habit (%)	87 (33.3%)	13 (25.0%)	52 (25.6%)
Smoking habit (%)	64 (24.5%)	12 (23.1%)	30 (14.7%) *

Values are means ± standard deviation of the demographic data. Values in () indicate percentage in each group. Differences among the non-OA, early knee OA, and radiographic knee OA groups were compared by analysis of variance or chi-square test. A *p* value below 0.05 when compared with non-OA (*) and early knee OA (†) were considered statistically significant.

*KOOS* The knee injury and osteoarthritis outcome scores, *ADL* Activities of daily livings, *QOL* Quality of life.

### Association between early knee OA and females hormones

**Figure 3:**
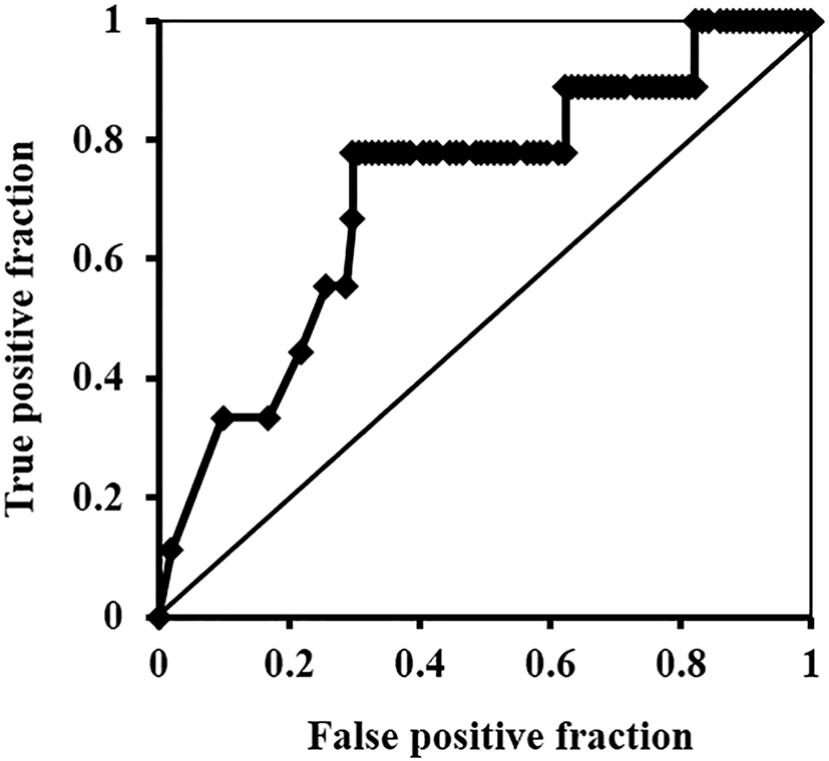
ROC curve of antimullerian hormone for presence of early knee osteoarthritis. The plot of false positive fraction and 1-true positive fraction build curve and area under the curve (AUC) was calculated as 0.712 (95%CI: 0.527–0.897, *p* = 0.024). The cut-off point was defined from the nearest point to the true positive, and estimated as 0.08 ng/ml.

Correlation between age and estradiol was weak (r = − 0.281, *p* = 0.002) at the pre-menopausal stage, and there was no correlation post-menopause (*p* = 0.334) (Fig. 2A). In contrast, the values of AMH in pre-menopausal females were 0.93 ± 1.22 ng/ml, which was highly correlated with age (r =  − 0.738; *p* < 0.001); the levels in post-menopausal females were 0.02 ± 0.01 ng/ml, regardless of age (*p* = 0.126) (Fig. 2B). Furthermore, the values of estradiol and AMH of early knee OA and radiographic knee OA were significantly lower than that of the non-OA group (Table 1). ROC analysis in pre-menopausal females with KL grade 0 and 1 showed that reduced serum levels of AMH reflected the presence of early knee OA, and its cutoff value was estimated at 0.08 ng/ml. Additionally, the odds ratio of AMH < 0.08 ng/ml for early knee OA was 8.28 (*p* = 0.024; AUC, 0.712; 95% CI, 0.527–0.897) (Fig. 3, Table 2). AUC of AMH was the highest among those of the other hormones: 0.636 in estradiol, 0.677 in LH, 0.697 in FSH, 0.513 in prolactin, and 0.513 in testosterone (Table 2). Based on the cutoff values of AMH, pre-menopausal females were grouped into two categories; the presence of early knee OA increased to 15.0%, compared with 3.5% in those with higher AMH (*p* < 0.001) (Table 3). Moreover, the AMHs were negatively correlated with bone absorption and formation markers (Table 4).

**Table 2:** ROC analysis of female hormones for early knee osteoarthritis.

Female hormone	AUC	95% CI	χ^2^ value	*p* value	Cut-off value	FPF	TPF	Odds ratio
Anti-Mullerian hormone	0.712	0.527–0.897	5.05	0.025	0.08	0.30	0.78	8.28
Estradiol	0.636	0.399–0.873	1.27	0.260	20.00	0.15	0.56	7.17
Luteinizing hormone	0.677	0.487–0.866	3.33	0.068	10.27	0.28	0.78	9.13
Follicle-stimulating hormone	0.697	0.509–0.886	4.22	0.040	9.65	0.30	0.78	8.28
Prolactin	0.513	0.286–0.740	0.01	0.909	16.32	0.18	0.33	2.31
Testosterone	0.513	0.325–0.702	0.02	0.890	0.21	0.33	0.44	1.62

ROC: AUC: area under the curve, 95% CI: 95% confidence interval, *FPF* False positive fraction, *TPF* True positive fraction.

**Table 3:** Prevalence of knee osteoarthritis in menopausal transition and post-menopausal stage.

	Pre-menopause		Post-menopause (%)
	AMH ≧ 0.08 (%)	AMH < 0.08 (%)	
Early knee osteoarthritis	3.5	15.0	10.9
Radiographic knee osteoarthritis	12.9	10.0	48.1

Prevalence of early and radiographic knee osteoarthritis in menopausal transition and post-menopausal stage. In pre-menopausal stage, subjects were divided into two groups according to the cut-off value (0.08 ng/ml).

**Table 4:** Correlation coefficients among biomarkers of female hormones, bone metabolism, and inflammation.

	BMD	AMH	E2	LH	FSH	Prolactin	Testosterone
AMH	0.491		− 0.458	− 0.555	0.595	0.185	0.127
BMD		0.491	0.496	− 0.224	− 0.334	0.186	0.102
Total P1NP	− 0.243	− 0.260	− 0.298	0.198	0.206	− 0.119	
BAP	− 0.365	− 0.356	− 0.363	0.243	0.261	− 0.163	
NTx	− 0.284	− 0.392	− 0.384	0.328	0.331		
TRACP-5b	− 0.465	− 0.506	− 0.479	0.351	0.410	− 0.211	− 0.105
Pentosidine	− 0.298	− 0.280	− 0.328	0.136	0.234	− 0.119	− 0.140
Homocysteine	− 0.214	− 0.152	− 0.175	0.141	0.175		
sHA	− 0.516	− 0.443	− 0.388	0.216	0.259	− 0.132	
MMP-3						0.095	0.132
Hs-CRP	− 0.131						
Adipokine	− 0.219	− 0.280	− 0.208	0.192	0.259		
Leptin		0.125	0.162	− 0.097	− 0.160		0.122
IL-6	− 0.244	− 0.155					

Spearman’s correlation coefficients were calculated. Only statistically significant correlations (*p* < 0.05) were shown in this table.

### Influence of bone metabolism on early knee OA and radiographic knee OA

Regarding bone fragility, the BMD of radiographic knee OA were lower than early knee OA and non-KOA (*p* < 0.001, respectively). Further, the values of BAP (*p* = 0.002), TRACP-5b (*p* = 0.002), and NTx (*p* < 0.001) of the radiographic knee OA group were significantly higher than those of the non-KOA group. Furthermore, the bone quality markers, pentosidine (*p* < 0.001), and homocysteine (*p* = 0.014) were worse in the radiographic knee OA group than in the non-KOA group. Bone metabolic and quality markers were negatively correlated with BMD, AMH, and estradiol concentration and positively correlated with LH and FSH (Table 4). Further comparison with inflammation markers showed that hyaluronan, MMP-3, leptin, and IL-6 concentrations in the radiographic knee OA group were higher than those in the non-KOA group. Hyaluronan moderately correlated with BMD (r =  − 0.516; *p* < 0.001), AMH (r =  − 0.443; *p* < 0.001), and estradiol (r =  − 0.388; *p* < 0.001) (Table 4).

### Related factors for early knee OA and radiographic knee OA from regression analysis

Models 1 and 2 logistic regression analysis focusing on AMH reduction at menopausal transition showed that the related AMH below 0.08 ng/ml was significantly related to early knee OA (*p* = 0.035; odds ratio, 5.55) and radiographic knee OA (*p* = 0.032; odds ratio, 1.59). In model 3, analysis focusing on the post-menopausal period showed that the risk of radiographic knee OA increased by 1.24 times every 5 years post-menopause (*p* = 0.005) (Table 5).

**Table 5:** Related factors for radiographic knee osteoarthritis.

	Standard partial regression coefficient	*p* value	Odds ratio	95% confidence interval
**Model 1: Risk for early knee OA in pre-menopausal females (Categorized AMH)**				
AMH reduction	1.71	0.035	5.55	1.13–27.23
Total PINP	− 0.05	0.106	0.95	0.90–1.01
Leptin	0.12	0.015	1.13	1.02–1.25
**Model 2: Risk for radiographic knee OA in overall females (Categorized AMH)**				
Body mass index	0.16	< 0.001	1.18	1.10–1.26
Bone mineral density	− 4.40	0.002	0.01	0.01–0.19
Hyaluronic acid	0.02	< 0.001	1.02	1.01–1.02
Total PINP	− 0.01	0.106	0.99	0.98–1.00
AMH reduction	0.46	0.032	1.59	1.04–2.42
**Model 3: Risk for radiographic knee OA in overall females (Postmenopausal period by 5 years)**				
Body mass index	0.16	< 0.001	1.17	1.10–1.25
Bone mineral density	− 2.70	0.086	0.07	0.01–1.47
Hyaluronic acid	0.01	< 0.001	1.01	1.01–1.02
Post-menopausal period	0.22	0.005	1.24	1.07–1.44

Logistic regression analysis by backward stepwise selection method was performed, with the presence of early knee osteoarthritis or knee osteoarthritis as dependent variable, and body mass index, bone mineral density, bone metabolic markers, and inflammation markers as independent variables. Furthermore, model 1 and 2 included categorized anti-Mullerian hormone (AMH) reduction (pre-menopause with AMH ≥ 0.08, pre-menopause with AMH < 0.08, or post-menopause). Model 3 included post-menopausal period by 5 years after menopause.

## Discussion

The most significant finding of this study was that reduced serum levels of AMH in middle-aged females were correlated with the presence of early knee OA. Furthermore, the cutoff value of serum AMH was estimated as 0.08 ng/ml from ROC analysis. In addition, reduced serum levels of both AMH and estradiol, which were proof of menopause, were correlated with lower BMD, higher turnover of bone metabolism, and increased inflammation. These results suggested that the decline of female hormone would track knee OA from the early stages. The causation between AMH and early knee OA was not concluded from cross-sectional cohort study; however, current study proved an apparent association between AMH and early knee OA, which indicates AMH might be one of the biomarkers for early knee OA in association with decline in female hormone, bone fragility, and inflammation. Further longitudinal analysis are needed to reveal the prognostic power of AMH as a biomarker for early knee OA.

Females are at a higher risk of knee OA, and many several epidemiological studies revealed that the prevalence of OA in females were higher than in males^7,26^. A systematic review estimated that the total odds ratio of females was 1.68 (95% CI, 1.37–2.07)^6^. Framingham study showed that the prevalence of OA in females was 36% and 1.2 times higher than that in males^11^. In Japan, the prevalence of OA in females were 1.5–2.8 times higher than in males^5,8,9^; the prevalence was higher than that of Caucasians. Considering the role played by menopause, a longitudinal study from Melbourne Women’s Midlife Health Project showed that those who have never undergone hormone therapy were at a higher risk of developing knee OA^27^. Further, representative therapy decreased the incidence of knee OA^28^. Although one systematic review concluded that there was no clear association between female hormonal aspects and knee OA^29^, the relationship between radiographic knee OA and menopause shown in this study is supported by previous studies.

This study could reveal the application of AMH as a serum biomarker to reflect the presence of knee OA, especially at an early phase of the disease. Until now, it is known that female hormones influence the etiology of knee OA, its precise monitoring was challenging, especially in menopausal transition. In these situations, AMH, an ovarian aging marker^18,19^, could reflect the presence of knee OA from the early phase. Although detailed direct mechanisms of AMH on knee OA have not revealed, ovarian aging led to the decrease of female hormones including estrogen. The longitudinal study would conclude the relationship among knee OA, menopause, and AMH. Despite this limitation, AMH had an advantage that it is not affected by menstrual cycles like estradiol, LH, and FSH, would be isolated biomarker for early knee OA.

Moreover, these results suggested that initiation of degenerative change was in line with the menopausal transition stage. In the early stages of knee OA, the Framingham study, including 710 knees with KL grade 0/1, showed the featured MRI findings in those with knee pain as having BMLs, attrition, and subchondral bone cysts^30^. Further, the CHECK study, a 5-year longitudinal cohort of general population, showed that the incidence risk factors for knee OA from KL grade 0/1 were BMLs, effusion, and meniscal lesion on MRI^31^. Bone abnormalities in the early stage of KL grade are likely to occur in the middle-aged female population^30,31^. During menopausal transition, bone metabolism changes dramatically, corresponding to the intrinsic hormonal changes in females. Based on this evidence, the current data indicates that such abrupt menopausal transition reflects not only increased systemic bone metabolism but also focal bone abnormalities, which could induce osteoarthritic changes in knee joints.

In this regard, both osteoporosis and knee OA are attributed to menopause^32,33^. However, the possibility of osteoporosis causing the development of knee OA is uncertain. Previous studies have reported that higher BMD is related to the incidence of knee OA^34^. Conversely, from the basis of evidences in the most recent decade, Ota et al. revealed that symptomatic BMLs on MRI were related to bone fragility, including lower BMD and high turnover of bone metabolism in middle-aged females without radiographic abnormalities^25^. Furthermore, others reported a pathogenic mechanism since excessive bone resorption takes place in the early phase of OA^35,36^. Previous reports support our results that lower BMD and increased bone metabolism of middle-aged females were correlated with the presence of knee OA. Indeed, osteoporotic osteoarthritis is reported as a particular type of OA and defined as OA showing a distinctive decrease in subchondral bone density accompanied by high remodeling rates^37,38^. Further studies are needed to establish the relationship between bone fragility and knee OA at any phase.

Another interesting result in this study was that serum AMH concentration in the female population having knee OA was associated with serum inflammation markers. Systemic and focal inflammation in the synovium play an important role even in the early phase of knee OA^39^. Inflammation is generally induced by microfragments of cartilages or danger-associated molecular pattern (DAMPS) in synovial fluids and releases several proteases and cytokines, which accelerate the degeneration of articular tissues^40–42^. The infrapatellar fat pad is a major source of adiponectin in synovial fluid; adiponectins are closely related to the metabolic syndrome and degenerative pathological changes in the cartilage and bone during OA^43^. In this study, the presence of early and radiographic knee OA correlated with an increasing serum hyaluronan concentration. Previous reports indicated that the serum levels of hyaluronan is considered as a biomarker for synovitis, which reflects the severity of knee OA and degree of pain^22,44^; additionally, this predicts future joint space narrowing over 5 years^45^. AMH was a valuable and predictive biomarker for early knee OA, which indicated bone fragility and inflammation. However, the utility and reliability of systemic biomarkers in OA diagnosis was not validated; further studies are needed.

This study had several limitations besides the selection bias focusing only on middle- to old-aged females. First, the study sample was the general Japanese population; their background information and confounding factors were not completely evaluated. Second, the imaging examination was limited to the radiographs. Although the MRI and ultrasonographic imaging detected minute structural changes and inflammation of the knee joint^21,31^ especially in early knee OA, we could not use these modalities. However, by measuring many inflammation biomarkers, we could evaluate the minute changes of osteoarthritis. Furthermore, regarding radiographic examination, patellofemoral joint OA was not fully examined. Although crepitus was evaluated as one of the symptoms of patellofemoral OA, an imaging analysis was not performed. Imaging of patellofemoral joints could not be performed as a result of time constraints. However, patellofemoral OA should be assessed because of its significant effect on ADL and QOL^46,47^. Third, BMD was measured only in the distal radius. Although it was ideal to evaluate the BMD of knee joint, it needs wide space and shield. In considering radiation exposure, it was difficult to measure the BMD of knee joint in the large sample cohort study. Finally, this cross-sectional cohort study could not conclude the causation between incidence of knee OA and reduced serum levels of AMH. Previously it is known that ovarian aging induces decreased female hormones, which affects the chondrocyte and subchondral bones. However, this has to be investigated by the longitudinal cohort study. Thus, a longitudinal observation was conducted in our cohort to estimate the real incidence of knee OA after AMH reduction and to validate its predictive values. Despite these limitations, this cohort study revealed the association of AMH and early knee OA during menopausal transition, in addition to the relationship between menopause and knee OA with lower BMD in middle-aged females. In considering the etiology of OA, Because serum levels of AMH decrease three years before menopause, which might reflect on knee OA via increasing bone metabolism, lower BMD, synovitis, or decreasing female hormones. Further longitudinal cohort study is needed.

## In conclusion

Reduced serum levels of AMH were correlated with the presence of early knee OA. AMH might be one of the biomarker for early knee OA. Also the AMH were correlated with the higher bone metabolic markers, lower bone mineral density, higher synovitis markers.

## Data Availability

All of data and material are available from the database of Department of Orthopaedic Surgery and Social Medicine, Hirosaki University Graduate School of Medicine.
